# Impact of cognitive reserve markers on incident mild cognitive impairment and dementia: Evidence from the Korean Longitudinal Study of Aging, 2006‐2022

**DOI:** 10.1002/alz70860_096465

**Published:** 2025-12-23

**Authors:** Hyun‐Ju Seo, Soon‐Ki Ahn, Min‐Jung Choi

**Affiliations:** ^1^ Chungnam National University, Daejeon, Daejeon, Korea, Republic of (South); ^2^ Chungnam National University Hospital, Jung‐gu, Daejeon, Korea, Republic of (South); ^3^ The Catholic University of Korea, Seoul, Korea, Republic of (South)

## Abstract

**Background:**

In South Korea, the annual incidence rate of dementia is 18.8 cases per 1,000 elderly individuals, rising with age from 12.5 cases per 1,000 (65–74 years) to 87.2 per 1,000 (≥85 years). In 2021, 22.7% of individuals aged 65 and older had mild cognitive impairment, indicating that one in five elderly individuals is affected. Dementia significantly contributes to functional limitation and dependency, underscoring the need for effective prevention and management strategies.

The purpose of this study is to identify population‐specific and modifiable cognitive reserve markers using Korean Longitudinal Study of Aging data collected prospectively from 2006 to 2020 in South Korea. Additionally, it aims to determine the magnitude of these markers as protective factors against the occurrence of mild cognitive impairment and dementia.

**Method:**

The study utilizes data from 10,254 individuals aged 45 and older who completed relevant questionnaires on cognitive reserve markers and mild cognitive impairment or dementia diagnoses in waves 7–9 (2018–2022), excluding those with prior diagnoses of mild cognitive impairment and dementia. Cognitive reserve indicators include education level, economic activity, type of occupation, leisure activities, physical activity, and financial management ability. Dementia incidence was self‐reported, with additional data on timing. Covariates include sociodemographic factors (age, sex, marital status, income) and modifiable risk factors (e.g., hypertension, depression).

**Result:**

Descriptive analyses will assess the incidence of dementia and mild cognitive impairment by cognitive reserve markers. Cox proportional hazards regression will estimate associations between cognitive reserve markers and the risk of dementia or mild cognitive impairment.

**Conclusion:**

This prospective cohort study will identify modifiable cognitive reserve markers and evaluate their protective effects, contributing to Korean‐specific brain health initiatives for dementia prevention and cognitive health promotion.

